# The Mesoscopic Connectome of the Cholinergic Pontomesencephalic Tegmentum

**DOI:** 10.3389/fnana.2022.843303

**Published:** 2022-05-17

**Authors:** Peilin Zhao, Huading Wang, Anan Li, Qingtao Sun, Tao Jiang, Xiangning Li, Hui Gong

**Affiliations:** ^1^Britton Chance Center for Biomedical Photonics, Wuhan National Laboratory for Optoelectronics, MoE Key Laboratory for Biomedical Photonics, Huazhong University of Science and Technology, Wuhan, China; ^2^HUST-Suzhou Institute for Brainsmatics, Jiangsu Industrial Technology Research Institute (JITRI), Suzhou, China

**Keywords:** cholinergic, pedunculopontine nucleus, laterodorsal tegmental nucleus, input, projection, whole brain

## Abstract

The pontomesencephalic tegmentum, comprising the pedunculopontine nucleus and laterodorsal tegmental nucleus, is involved in various functions *via* complex connections; however, the organizational structure of these circuits in the whole brain is not entirely clear. Here, combining viral tracing with fluorescent micro-optical sectional tomography, we comprehensively investigated the input and output circuits of two cholinergic subregions in a continuous whole-brain dataset. We found that these nuclei receive abundant input with similar spatial distributions but with different quantitative measures and acquire similar neuromodulatory afferents from the ascending reticular activation system. Meanwhile, these cholinergic nuclei project to similar targeting areas throughout multiple brain regions and have different spatial preferences in 3D. Moreover, some cholinergic connections are unidirectional, including projections from the pedunculopontine nucleus and laterodorsal tegmental nucleus to the ventral posterior complex of the thalamus, and have different impacts on locomotion and anxiety. These results reveal the integrated cholinergic connectome of the midbrain, thus improving the present understanding of the organizational structure of the pontine-tegmental cholinergic system from its anatomical structure to its functional modulation.

## Introduction

The pontomesencephalic tegmentum (PMT) is a heterogeneous nucleus containing cholinergic, GABAergic, and glutamatergic neurons located at the junction of the midbrain and the pons (Wang and Morales, 2009). As an indispensable part of the ascending reticular activation system, PMT cholinergic neurons are important converters of the sleep-wake state that mainly regulates the conversion between REM sleep and non-REM sleep (Van Dort et al., 2015). As the major source of acetylcholine in the midbrain dopaminergic (DA) system, PMT cholinergic neurons govern the activity of DA neurons in the ventral tegmental area (VTA) and are involved in reward (Steidl et al., 2017; Zhang et al., 2018) and addiction (Picciotto and Corrigall, 2002; Schmidt et al., 2009). Meanwhile, various studies suggest that PMT cholinergic neurons contribute to locomotion (Dobbs and Cunningham, 2014; Dautan et al., 2016), and their numbers decrease significantly in some neurodegenerative diseases with movement disorders, such as Parkinson’s disease and dementia with Lewy bodies (Pepeu and Grazia Giovannini, 2017). In addition, PMT cholinergic neurons participate in epilepsy pathology (Soares et al., 2018).

According to the distribution of cholinergic neurons, the PMT is anatomically divided into the pedunculopontine nucleus (PPN), which is located in the anterior part, and the laterodorsal tegmental nucleus (LDT), which is located in the posterior part (Mesulam et al., 1983). Previous studies have certified the functional difference between these two components of the pontine-tegmental cholinergic system (ChAT^PPN^ and ChAT^LDT^). Apart from reward, ChAT^LDT^ neurons are also involved in stress-induced depressive-like behaviors (Fernandez et al., 2018) and food consumption (Dickson et al., 2010) *via* the VTA circuits. ChAT^PPN^ but not ChAT^LDT^ neurons mediate ketamine-induced prefrontal serotonin release from the dorsal raphe nucleus (DR) (Kinoshita et al., 2018) and regulate locomotion by projecting to the ventral substantia nigra pars compacta (vSNc) (Xiao et al., 2016).

The multiple functions of PMT cholinergic neurons are based on their complex connections, containing local and long-range circuits in the whole brain (Martinez-Gonzalez et al., 2011; Mena-Segovia and Bolam, 2017). To reveal the anatomical mechanism underlying the functional difference, it is critical to comprehensively investigate the organizational structure of these cholinergic nuclei. Recent studies have described the afferent or projection circuit from different aspects, such as the input circuits of PMT cholinergic neurons in mice (Wang et al., 2019; Henrich et al., 2020) and rats (Huerta-Ocampo et al., 2021) and the projection patterns of ChAT^PPN^ (Henrich et al., 2020). These results suggest that ChAT^PPN^ and ChAT^LDT^ neurons receive similar afferents with quantitative differences and have different projecting preferences in some regions, such as the thalamus (Huerta-Ocampo et al., 2020; Sokhadze et al., 2021), striatum, and substantia nigra (Mena-Segovia and Bolam, 2017). However, these studies mainly focused on special regions and mapped the circuits on serial brain slices; therefore, the spatial distribution and continuous circuits in the whole brain were not assessed. To deeply understand the complex connections of these two nuclei, we need a comprehensive understanding of their input and output circuits, particularly the long-range connections with different brain regions.

Herein, combining the virus tracing system with fluorescence micro-optical sectioning tomography (fMOST) serial technologies (Gong et al., 2016), we uncovered the long-range connectome of PMT cholinergic neurons with high resolution and decoded the diverse unidirectional cholinergic projections from the PPN and LDT to the ventral posterior complex of the thalamus (VP) and certified their different roles *via* optogenetics.

## Materials and Methods

### Animals

Adult (2–4 months) ChAT-ires-Cre mice, Ai14 reporter mice, and C57BL/6J mice were used in this study. ChAT-Cre transgenic mice (stock No: 018957) and Ai14 reporter mice (stock No: 007914) were purchased from Jackson Laboratory. We employed ChAT-Cre mice to trace the input and output circuits. The C57BL/6J mice were used for control experiments and retrograde tracing. The mice were kept under a condition of a 12-h light/dark cycle with food and water *ad libitum*. All animal experiments were approved by the Animal Care and Use Committee of Huazhong University of Science and Technology.

### Virus and Tracer Information

For input tracing, two adeno-associated viruses (AAV) helpers, rAAV2/9-Ef1α-DIO-RG and rAAV2/9-Ef1α-DIO-His-BFP-TVA [both 2–5 × 10^12^ genome copies (gc)/ml], and one modified rabies virus (RV), RV-EnvA-DG-GFP [2.5 × 10^8^ international units (IU)/ml], were used. rAAV- Ef1α-DIO-EGFP (2–5 × 10^12^ gc/ml) was used for output tracing. AAV-DIO-ChR2-mCherry and AAV-DIO-mCherry (2–5 × 10^12^ gc/ml) were used for optogenetics activation. rAAV-hSyn-retro-cre (2–2.5 × 10^12^ gc/ml) was injected in Ai14 mice. All the viruses were purchased from BrainVTA (BrainVTA Co., Ltd., Wuhan, China). We also employed the 2% CTB 647 (Thermo Fisher, Waltham, MA, United States, C34778), 2% FG (Biotium, 52-9400, San Francisco Bay Area, United States), and RetroBeads (Lumafluor, R180-100, Carlisle, England) for retrograde tracing.

### Surgery and Viral Injection

Before virus injection, we anesthetized the mice with mixed anesthetics (2% chloral hydrate and 10% ethylurethane dissolved in 0.9% NaCl saline) according to their body weight (0.1 ml/10 g). The heads of anesthetized mice were fixed with a stereotaxic holder to adjust the position of skulls. Then, a cranial drill (a ∼0.5-mm diameter) was employed to drill a hole above the target area.

The sterotaxic coordinates of nuclei are relative to Bregma. For input information acquisition, 75–100 nl of AAV helper (mixed with rAAV2/9-Ef1α-DIO-oRVG and rAAV2/9-Ef1α-DIO-His-BFP-TVA with a ratio of 1:1) was injected into the PPN (AP, −4.6 mm; ML, −1.35 mm; DV, −4 mm) and LDT (AP, −5.15 mm; ML, −0.5 mm; DV, −3.4 mm) with speed at 20 nl/min. Following the completion of viral injection, the needle was held for 10 min at the site and then retreated slowly. After that, incisions were stitched, and lincomycin hydrochloride and lidocaine hydrochloride gel was applied to prevent inflammation and alleviate pain for the animals. Three weeks later, 200–250 nl of RV-EnvA-DG-GFP was injected into the same location using the same methods mentioned above. For output tracing, 50 nl rAAV- Ef1α-DIO-EYFP was injected into the PPN and LDT of ChAT-Cre mice.

For optogenetics, 200–250 nl AAV-DIO-ChR2-mCherry or AAV-DIO-mCherry was injected into the PPN or LDT of ChAT-Cre mice. After virus injection, optical fibers (diameter, 200 μm, NA = 0.37) were planted into the VP (AP, −1.75 mm; ML, −1.7 mm; DV, −3.35 mm) of ChAT-Cre mice. The dental cement and skull screws were applied to fix the optical fibers for further experiments.

### Histology and Immunostaining

The histological operations were similar with the previous studies (Zhao et al., 2020). Four weeks after virus injection, anesthetized mice were perfused with 0.01-M PBS (Sigma-Aldrich, St. Louis, MO, United States), followed with 2.5% sucrose and 4% paraformaldehyde (PFA, Sigma-Aldrich, St. Louis, MO, United States), dissolving in 0.01-M PBS. The brains were removed and post-fixed in 4% PFA solution overnight.

For immunostaining, brain samples were sectioned at 50-μm coronal slices with the vibrating slicer (VT 1200S, Leica, Wetzlar, Germany). Every-second sections containing the PPN or LDT were selected for signal detection and neuron identification at the injection site, and other sections containing VTA, DR, and LC were selected to verify the cell types of monosynaptic inputs. These sections were blocked with 0.01-M PBS containing 5% (wt/vol) bovine serum albumin (BSA) and 0.3% Triton X-100 for 1 h at 37°C. Then, the sections were incubated with primary antibodies (12 h at 4°C): anti-ChAT (1:500, goat, Sigma-Aldrich, St. Louis, MO, Unites States, AB144P), anti-Tyrosine Hydroxylase (1:1,000, rabbit, Sigma-Aldrich, St. Louis, MO, United States, T8700), and anti-Tryptophan hydroxylase 2 (1:1,000, rabbit, Invitrogen, Carlsbad, CA, United States, PA1-778). Then, the sections were washed in PBS 5 times (5 min each) at room temperature. Next, these sections were incubated with fluorophore-conjugated secondary antibodies (2 h at 37°C): Alexa-Fluor 647, donkey anti-goat; Alexa-Fluor 647, donkey anti-rabbit (1:500, Abcam, Cambridge, MA, United States). After rinsing with PBS, DAPI (1 ng/ml) was applied on stained sections for 5 min, and the sections were finally mounted on glass slides with 50% glycerol after rinsing. We acquired the images of the sections with the confocal microscope (LSM 710, Zeiss, Jena, Germany).

### Imaging and 3D Visualization

Virus-labeled samples were dehydrated with alcohol and embedded with resin in preparation for whole-brain imaging. The whole-brain datasets were obtained with the fMOST system. First, we secured the embedded sample on the base of the imaging platform. Then, the top surface of the sample was imaged in two channels simultaneously. The imaged top surface was subsequently removed by a diamond knife. Thus, we could obtain a continuous whole brain dataset layer by a layer with high resolution (0.32 μm × 0.32 μm × 2 μm).

For 3D visualization and statistical analysis of whole brain datasets, we registered the whole-brain datasets to the Allen CCFv3. The detailed registration protocols have been described elsewhere (Ni et al., 2020). Briefly, uneven illumination was corrected, and background noises were removed by image processing. The down sampled data (the voxel resolution of 10 μm^3^) were uploaded into Amira software (v6.1.1, FEI, Mérignac Cedex, France) to distinguish and extract regional features of 15 anatomical invariants, including the outline of the brain, the ventricles, the corpus callosum, and the corpus striatum, etc. Next, the current advanced gray-level-based registration algorithm was employed to register the extracted features and obtain corresponding relationships between the image dataset and the Allen CCFv3 brain atlas. Basic operations, including extraction of areas of interest, resampling, and maximum projection, were performed *via* Amira software and Fiji (NIH).

### Quantification and Statistical Analysis

For cell counting, we acquired the spatial information of all the labeled neurons from the whole-brain data by NeuroGPS software (Quan et al., 2013) and registered the datasets to Allen CCFv3 using the methods mentioned above. Then, we quantified the number of input cells in each individual nucleus.

For output, the process for quantification has been described before (Xu et al., 2021). Briefly, the datasets at the voxel resolution of 10 μm^3^ were registered to the Allen CCFv3 and subsequently performed on the original data to acquire registered datasets at the voxel resolution of 1 μm^3^. Then, we recut the registered datasets and performed with binary processing to extract the labeled signals. Next, we checked the extracted signals with original data and removed the noise information. Finally, we obtained output in different nuclei by splitting the whole-brain dataset into small volumes of 1 μm × 1 μm × 1 μm and extracting signals from each cube.

For statistical analysis, the number of input neurons in discrete brain regions was normalized to the total number of input neurons, and the output in different nuclei was normalized to the total output signals. The signals of the injection site were excluded. Statistical graphs were generated using GraphPad Prism v.8.02 and Microsoft Excel (Office 2020). We employed GraphPad Prism v. 8.02 to compare the significant differences. We conducted two-sided *t*-tests to compare the difference between the percentages of input neurons or output fibers of the PPN and LDT from each valid nucleus (Huerta-Ocampo et al., 2021). We compared the behavioral results using paired *t*-tests. The confidence level was set to 0.05 (*P* value), and all results were presented as mean ± SEM. We employed SPSS (IBM SPSS) for cluster analysis.

### Open Field Test

To explore the influence of PMT cholinergic neurons on the locomotion, we employed the open field test (Kraeuter et al., 2019) to value the effect of optical activation of the ChAT^PPN^-VP or ChAT^LDT^-VP circuits. The open field consisted of a square arena (40 cm × 40 cm × 40 cm) with ivory-white walls. The USB-connected camera was set 150 cm above the floor of the arena. EthoVision XT 12.0 (Noldus Apparatus) was used to track the animal and analyze the total traveled distance of the animals. On Day 1 to Day 3, all the animals explored the arena freely for 10 min for habituation. On Day 4, the animals were allowed to explore the arena for 6 min (3 min without light followed by 3-min light stimulation).

### Elevated Plus Maze

To evaluate the potential influence on anxiety resulting from the activation of the ChAT^PPN^-VP or ChAT^LDT^-VP circuits, ChR2-infected ChAT-Cre mice were tested in the elevated plus maze (Tovote et al., 2015) (the open arm, 25 cm × 5 cm; the close arm, 25 cm × 5 cm × 15 cm; the center, 5 cm × 5 cm). In this experiment, the mice were placed in the center area with their heads toward the close arm. When the mice explored the open arm, we started to record the movement of the mice for 6 min (3 min free moving without light followed by 3-min light stimulation).

## Results

### Whole-Brain Input to Cholinergic Neurons in the Pontomesencephalic Tegmentum

**FIGURE 1:**
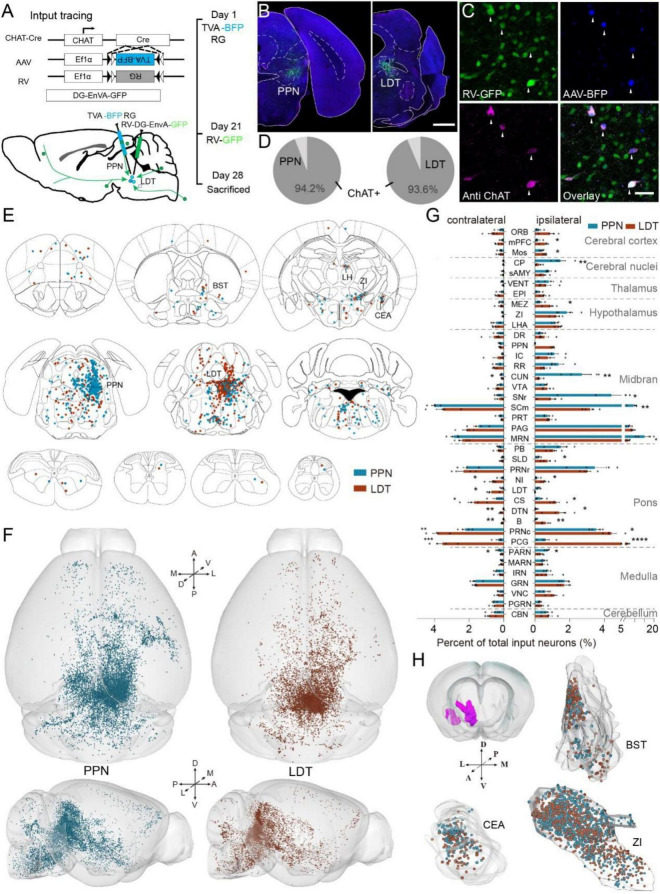
Whole-brain input of pontomesencephalic tegmentum (PMT) cholinergic neurons. **(A)** The methods and processes for monosynaptic input tracing. **(B)** A typical coronal plane of an inject site. **(C)** Immunofluorescent staining of start cells. GFP-labeled neurons are input neurons. BFP-labeled neurons are infected by an AAV helper. RFP represents cholinergic neurons. The neurons co-labeled with BFP, GFP, and RFP are start cells; arrows point out start cells. **(D)** The proportion of ChAT-positive start cells, PPN = LDT = 4 mice. **(E)** Input neurons of ChAT^PPN^ and ChAT^LDT^ distribute in the whole-brain widely. **(F)** The 3D view of whole brain input neurons. Left displays the whole-brain input to the PPN; right represents LDT. **(G)** Quantitative statistical proportion of main input regions to whole-brain input neurons. The left side of vertical axis represents proportion of input neurons in the contralateral hemispheres, and the right side represents ipsilateral hemispheres. **(H)** The spatial distribution of input neurons in the BST, CEA, and ZI. A scale bar, **(B)** 1,000 μm; **(C)** 50 μm. The abbreviations of brain regions are provided in Supplementary Table 1. Data are shown as mean ± SEM. Two-sided *t*-tests, *p < 0.05, **p < 0.01, ***p < 0.001, ****p < 0.0001; PPN = LDT = 4 mice; for detailed P values, see Supplementary Table 2.

To obtain afferent information, a Cre-dependent virus tracing system was employed on ChAT-ires-Cre mice. First, we performed two Cre-dependent adeno-associated virus (rAAV2/9-Ef1α-DIO-RG and rAAV2/9-Ef1α-DIO-His-BFP-TVA) on the ChAT-Cre mice to coexpress TVA receptors and G proteins in cholinergic neurons (Figure 1A). Three weeks later, we injected the modified rabies virus (RV) in the same position. The modified RV (RV-DG-EnvA-GFP) was encapsulated in EnvA proteins, which could only infect cholinergic neurons, expressing TVA receptors and then transsynaptically labeled monosynaptic input neurons with the help of G protein (Wall et al., 2010). The neurons colabeled with BFP and GFP in the injection site were start cells, while the neurons only labeled with GFP were input neurons (Figures 1B,C). To evaluate the specificity of the virus used, we verified the start cells with immunofluorescent staining and found that more than 93% of start cells (4 mice; PPN, 93.9 ± 1.2%; LDT, 93.5 ± 1.3%) were ChAT-positive neurons (Figures 1C,D). Then, we performed RV on the C57BL/6J mice directly and found two neurons in the injected site of the two mice (Supplementary Figures 1A–E). Finally, we injected AAV helpers and subsequently RV into the PPN of C57BL/6J mice, and only several neurons in the injection site were labeled, while no neurons were infected in other input areas (Supplementary Figures 1F–I). These results suggest that the virus we used has good specificity, and that long-range labeled neurons are input neurons of cholinergic neurons. As previously reported (Watabe-Uchida et al., 2012), leakage of virus would result in putative overestimation of local presynaptic cells. Herein, we only analyzed long-range input neurons in the present study.

Consistent with previous results (Wang et al., 2019; Henrich et al., 2020), ChAT^PPN^ and ChAT^LDT^ received monosynaptic afferents from similar regions in the whole brain (Figure 1E and Supplementary Figure 2A). The afferent neurons were dispersed throughout the whole brain and clustered in some nuclei, such as the bed nucleus of the stria terminalis (BST), central amygdaloid nucleus (CEA), and zona incerta (ZI). In addition, monosynaptic input neurons were also present in the cerebellum and spinal cord; those neurons gathered in three nuclei of the vermis cerebelli and dispersed in the cervical, thoracic, lumbar, and sacral vertebrae in the spinal cord (Figure 1E and Supplementary Figure 2A).

To compare the input patterns, we embedded the RV-labeled brains (PPN = LDT = 4 mice) with resin and obtained continuous three-dimensional datasets with high resolution at 0.32 μm × 0.32 μm × 2 μm *via* fMOST (Gong et al., 2016). We acquired the spatial information of input neurons *via* NeuroGPS software (Quan et al., 2013) and then registered the data to the Allen Mouse Brain Common Coordinate Framework version 3 (Allen CCFv3) (Wang et al., 2020; Figure 1F). 3D data showed that input neurons were widely distributed in the bilateral hemispheres. To analyze the input circuits accurately, we counted all the input neurons in the bilateral hemispheres and calculated the proportion of total inputs (Figure 1G and Supplementary Figure 2B). We acquired all the long-range input neurons (PPN, 1,8052 ± 5,024 neurons; LDT, 15,436 ± 2,036 neurons; PPN = LDT = 4 mice) and found that most of them were located in the ipsilateral hemisphere (PPN, 75.05 ± 1.54%; LDT, 64.70 ± 1.34%). The input neurons were mainly located in the midbrain (PPN, 55.84 ± 1.98%; LDT, 37.45 ± 1.69%) and pons (PPN, 17.48 ± 1.22%; LDT, 34.67 ± 1.14%), followed by the medulla and hypothalamus. To evaluate the consistency of the inputs to the same groups, we employed Pearson’s correlation analysis (Supplementary Figure 2C). We found good consistency among different samples in each group, with a correlation coefficient above 0.89, which demonstrated the consistency and reliability of our data.

Then, we compared the proportion of the main input nuclei. As shown in Figure 1G, the PPN was preferred by some nuclei, contributing to locomotion (White, 2009; Caggiano et al., 2018; Grillner and El Manira, 2020; Liu et al., 2020), including the caudoputamen (CP), the reticular part of the substantia nigra (SNr), SCm, midbrain reticular nucleus (MRN), cuneiform nucleus (CUN), and parvicellular reticular nucleus (PARN). Meanwhile, LDT received more input from some nuclei in the pons and limbic cortical areas, including the medial prefrontal cortex (mPFC), secondary motor area (MOs), sublaterodorsal nucleus (SLD), contralateral LDT, nucleus incertus (NI), superior central nucleus raphe (CS), dorsal tegmental nucleus (DTN), Barrington’s nucleus (B), pontine central gray (PCG), and the caudal part of pontine reticular nucleus (PRNc). In addition, ChAT^LDT^ also received stronger input from the ipsilateral hypothalamic medial zone (MEZ) (*P* values presented in Supplementary Table 2). We also valued the spatial distribution of input neurons in some similar input regions. As shown in Figure 1H, we extracted the spatial information of the cell body in the BST, CEA, and ZI and placed it in the same outline. We found that the input neurons of the PPN and LDT in these nuclei were mixed.

These results decode the continuous input atlas of two groups of PMT cholinergic neurons. We found that they receive afferents from the same brain regions but with quantitative differences. The ChAT^PPN^ receives richer input from nuclei involved in locomotion, while the ChAT^LDT^ is preferred by some nuclei in the pons and limbic cortical areas. Meanwhile, both of them accept steady input from the cerebellum and spinal cord.

### Neuromodulatory Characterization of Monosynaptic Afferents

**FIGURE 2:**
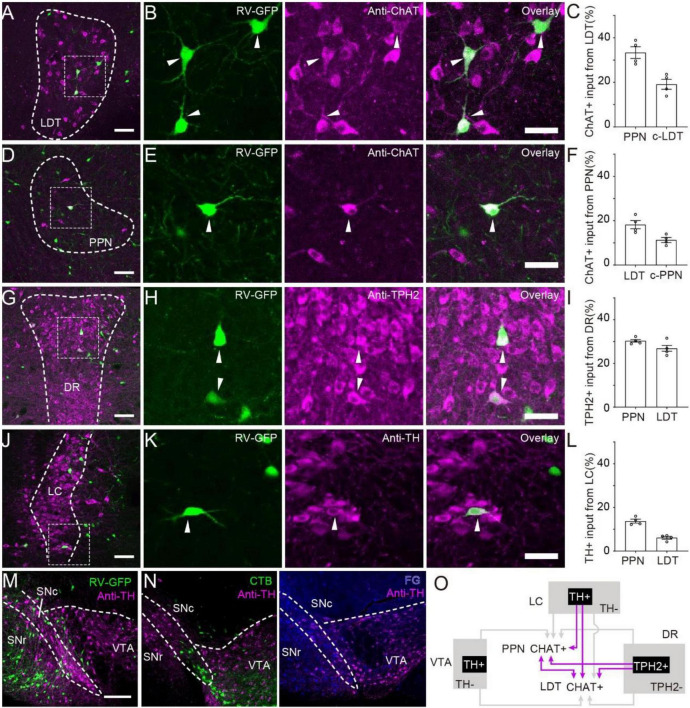
Monosynaptic afferent from neuromodulator neurons. **(A)** Immunohistochemical staining of input from LDT to PPN. **(B)** A three-panel presentation with RV, anti-ChAT, and overlay; arrows point out ChAT-positive input neurons. **(C)** Proportion of ChAT+ neurons input from LDT to PPN. **(D)** Immunohistochemical staining of input from PPN to LDT. **(E)** A three-panel presentation with RV, anti-ChAT, and overlay; arrows point out ChAT-positive input neurons. **(F)** Proportion of ChAT-positive neurons input from PPN to LDT. **(G)** Immunohistochemical staining of input neurons in the DR. **(H)** A three-panel presentation with RV, anti-TPH2, and overlay; arrows point out TPH2-positive input neurons. **(I)** Proportion of TPH2+ neurons input from DR to PPN or LDT. **(J)** Immunohistochemical staining of input neurons in the LC. **(K)** A three-panel presentation with RV, anti-TH, and overlay; arrows point out TH-positive input neurons. **(L)** Proportion of TPH2+ neurons input from DR to PPN or LDT. **(M,N)** Immunohistochemical staining of input neurons in the VTA labeled with RV, CTB, and FG, respectively. **(O)** The interconnections between PMT cholinergic neurons and neuromodulatory nuclei. The abbreviations of nuclei suffixed with “-c” meant nuclei in the contralateral. A scale bar − **(A,D,G,J,M,N)** 200 μm; **(B,E,H,K)** 50 μm. Data are shown as mean ± SEM. PPN = LDT = 4 mice.

Neuromodulator neurons in the brainstem containing cholinergic neurons in the PMT, dopaminergic neurons in the VTA and SNc, serotonergic neurons in the DR and noradrenergic neurons in the locus coeruleus (LC) are essential for the ascending reticular activation system to participate in multiple functions (Jones, 2003). Previous studies have verified that PMT cholinergic neurons modulate the activity of neuromodulator-releasing neurons in the VTA, SNc (Xiao et al., 2016), and DR (Kinoshita et al., 2018). Our results showed that both the PPN and LDT received input from these nuclei. To investigate the monosynaptic afferent from neuromodulator neurons to the PMT, we verified the molecular phenotypes of RV-labeled neurons *via* immunofluorescence staining (Figure 2). First, we investigated the interconnections of the PPN and LDT (Figures 2A–F). We found that cholinergic cells in the PPN and LDT stably interacted with each other (LDT to PPN, 33.4 ± 2.6%; PPN to LDT, 18.3 ± 1.8%). Meanwhile, the PPN and LDT received input from contralateral homogeneous nuclei, and we found that the cholinergic neurons in the bilateral areas interconnected with each other steadily (PPN to PPN, 11.3 ± 1.1%; LDT to LDT, 19.2 ± 2.3%). In the DR, we found that more than a quarter of input neurons expressed tryptophan hydroxylase (TPH2), a biomarker for serotoninergic neurons (PPN, 30.7 ± 1.7%; LDT 27.7 ± 3.9%) (Figures 2G–I). In the LC, immunofluorescence staining showed that a few input neurons were TH-positive (PPN, 14.1 ± 1.9%; LDT, 5.7 ± 1.4%) (Figures 2J–L).

The VTA and SNc are central nuclei of the DA system in the midbrain and are filled with dense DA neurons. However, none of the RV-labeled neurons were co-labeled with TH (Figure 2M). Previous studies detected a direct regulation of cholinergic neurons in the PPN by DA neurons in the SNc in rats (Ryczko et al., 2016). Some studies have shown that modified RV may transsynaptically infect DA neurons weakly (Wall et al., 2013). To confirm the results, we labeled the input circuits of the PPN and LDT with cholera toxin B subunit (CTB) and fluorogold (FG) (Supplementary Figure 3A). Many neurons were found in the VTA, but none of them were TH-positive neurons (Figure 2N and Supplementary Figure 3B). Although previous studies have indicated that limitations of TH staining are not detectable in some conditions (Dautan et al., 2021), DA neurons in the midbrain projected most of their fibers ascending to the striatum, while a few projected to descending areas (Beier et al., 2015). In view of the abundant input neurons from the VTA, it is likely that different types of neurons in the VTA directly modulate PMT cholinergic neurons, but most of them are non-DAergic neurons.

Overall, ChAT^PPN^ and ChAT^LDT^ neurons steadily interconnected with each other, and both received similar and stable afferents from neuromodulator neurons in the DR and LC (Figure 2O).

### Whole-Brain Projection Pattern

**FIGURE 3:**
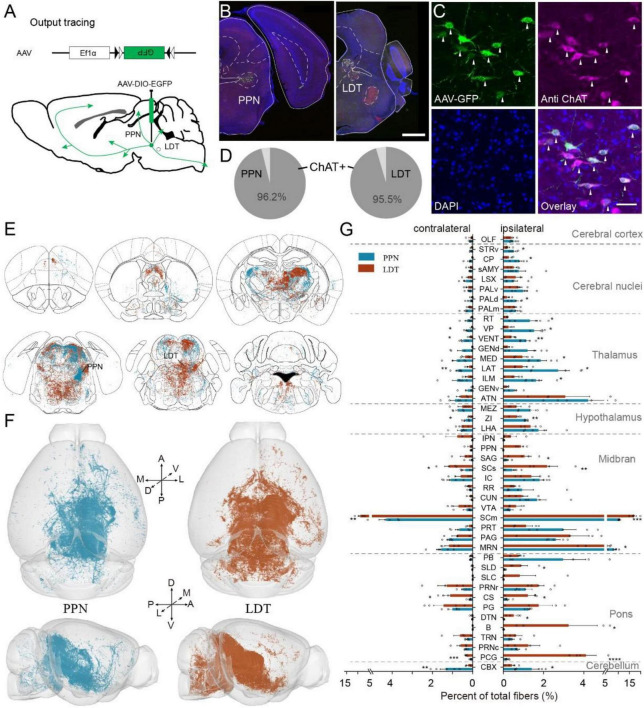
Whole brain projection of PMT cholinergic neurons. **(A)** The methods and processes for output tracing. **(B)** A typical coronal plane of an inject site; labeled cholinergic neurons are limited in object regions. **(C)** Immunofluorescent staining of cholinergic neurons; arrows point out ChAT-positive neurons. **(D)** The proportion of ChAT-positive neurons, PPN, 3 mice; LDT, 3 mice. **(E)** The whole-brain projection of PMT cholinergic neurons. **(F)** The 3D view of cholinergic projections in the whole brain. Left figures display the whole-brain projection of the PPN; right represents LDT. **(G)** Quantitative statistical proportion of main targeting regions from PPN and LDT. The left side of vertical axis represents proportion of cholinergic fibers in the contralateral hemispheres, while the right side represents ipsilateral hemispheres. A scale bar, (B) 1,000 μm; **(C)** 50 μm. The abbreviations of brain regions are provided in Supplementary Table 1. Data are shown as mean ± SEM. Two-sided *t*-tests, *p < 0.05, **p < 0.01, ***p < 0.001, ****p < 0.0001; PPN, 5 mice; LDT, 4 mice. For detailed P values, see Supplementary Table 2.

The ChAT^PPN^ and ChAT^LDT^ neurons integrated abundant input information from the whole brain and delivered it to various regions. To explore the difference between the efferent connection of the PPN and LDT, we traced the cholinergic projection in the whole brain with Cre-dependent virus expressing GFP (AAV-DIO-EGFP) in ChAT-Cre mice (Figures 3A,B). More than 95% of labeled neurons were ChAT-positive (3 mice each; PPN, 96.3 ± 1.1%; LDT, 95.4 ± 0.8%) (Figures 3C,D). Similar to previous studies (Henrich et al., 2020), cholinergic fibers from the PPN were widely distributed in different regions from the telencephalon to medulla, and abundant fibers reached the contralateral hemisphere. The ChAT^LDT^ neurons also sent rich fibers to the whole brain (Figure 3E and Supplementary Figure 4A). As shown in Figure 3E, ascending fibers projected throughout the midbrain, thalamus, hypothalamus, and basal forebrain and even to cortical areas, while descending fibers filled in the pons, medulla, and cerebellum.

To investigate the output information accurately, we embedded the labeled samples with HM20 resin and acquired whole-brain datasets at a resolution of 0.32 μm × 0.32 μm × 1 μm *via* fMOST (Figure 3F; PPN, 5 mice; LDT, 4 mice). Then, we quantified the output in the bilateral hemispheres and calculated the proportion of total output, following a previous study (Xu et al., 2021; Figure 3G and Supplementary Figure 4B). For the PPN, most cholinergic fibers were present in the midbrain (41.36 ± 1.05%), followed by the thalamus (22.59 ± 2.58%). The LDT also extended most fibers in the midbrain (51.84 ± 5.23%) but subsequently in the pons (23.34 ± 2.84%). To ensure the validity of our datasets, we evaluated the data *via* Pearson’s correlation, and the results suggested good consistency of different samples in each group, with a correlation coefficient above 0.81 (Supplementary Figure 4C). Then, we compared the main targets of PPN or LDT to compare their differences. As shown in Figure 3G, the ChAT^PPN^ neurons sent richer fibers to multiple ascending areas and the cerebellar cortex (CBX), including the dorsal region of the pallidum (PALd), CP, striatum ventral region (STRv), ZI, lateral and ventral groups of the dorsal thalamus (LAT, VENT), VP, intralaminar nuclei and medial regions of the dorsal thalamus (ILM, MED), reticular nucleus of the thalamus (RT), and MRN. The ChAT^LDT^ extended stronger fibers to some targets in the midbrain and pons, including the sensory-related superior colliculus (SCs), SCm, superior central nucleus raphe (CS) and pontine central gray (PCG), nucleus sagulum (SAG), SLD, dorsal tegmental nucleus (DTN), and Barrington’s nucleus (B) (detailed *P* values are presented in Supplementary Table 2).

In general, the PPN and LDT have similar and extensive cholinergic projections in the whole brain. ChAT^PPN^ neurons prefer some ascending targets, while ChAT^LDT^ neurons extend more fibers to the midbrain and pons.

### Connection Pattern of Long-Range Input and Output Circuits

**FIGURE 4:**
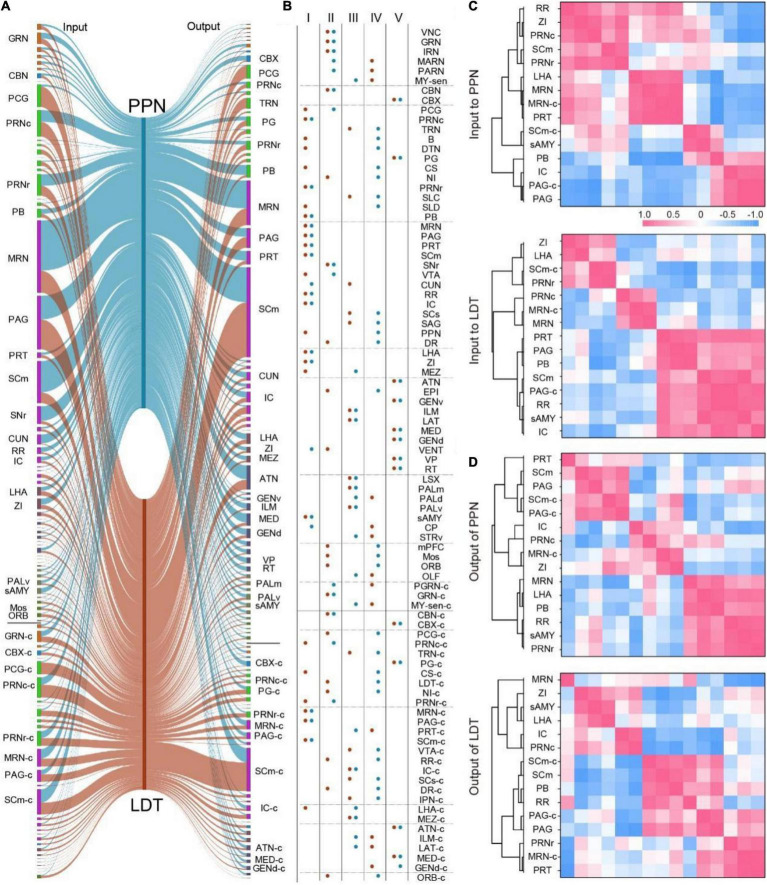
The connectivity pattern of input and output circuits. **(A)** The input and output relation of 88 major connecting nuclei of ChAT^PPN^ and ChAT^LDT^. The line width represents relative proportion. Nuclei with the same color belong to same regions. The abbreviations of nuclei suffixed with “-c” meant nuclei in the contralateral. **(B)** The major connecting nuclei were divided into five groups according to the input and output relation with ChAT^PPN^ or ChAT^LDT^. The connections with PPN and LDT were displayed with blue and red, respectively. **(C,D)** Clustering analysis of 15 nuclei that have strong interconnections with ChAT^PPN^ and ChAT^LDT^ simultaneously according to the Pearson’s correlation. The abbreviations of brain regions are provided in Supplementary Table 1.

According to the input and output atlas, we found that most nuclei projecting to the PMT also received cholinergic fibers from the PMT (Figures 1F, 3F). To investigate the long-range connectome clearly, we compared the connection between the PMT and 88 major connecting nuclei (input or output ≥ 0.5%) bilaterally (Figure 4A). As shown in Figure 4B, these nuclei had diverse interconnection patterns with ChAT^PPN^ neurons or ChAT^LDT^ neurons, such as strong interconnection (Type I), abundant input but sparse output (Type II), and sparse input but rich output (Type III). Type I nuclei are important regions that exchange information with the PPN or LDT and mainly from the midbrain, pons, and hypothalamus, such as the PRNc, SCm, and ZI. Type II nuclei are important afferent regions for the PPN or LDT and gather in the midbrain, including the VNC and IRN. Type III nuclei were important targets for information dissemination of the PPN or LDT and concentrated in ascending areas, including the thalamus and cerebral nuclei.

We also found that some of these nuclei had weak connections with one but strong connection with the other (Type IV) in the connection with PPN and LDT. They were the main differentially connected nuclei of the PPN and LDT. In addition, our results suggested that some nuclei received abundant cholinergic fibers from the PPN and LDT unidirectionally (Type V). They are important targets for transferring information from the pontine-tegmental cholinergic system and are mainly located in the thalamus.

As shown in Type I (Figure 4B), there were 15 nuclei that had strong interconnections with the PPN and LDT simultaneously. Then, we performed correlation analysis and hierarchical cluster analysis on these nuclei to investigate the similarities and variances of afferent and efferent nuclei (Figures 4C,D and Supplementary Figure 5). As a result, the clusters were diverse for the inputs or outputs of ChAT^PPN^ neurons and ChAT^LDT^ neurons. Regarding input circuits (Figure 4C and Supplementary Figure 5A), afferent regions gathered in 3–4 clusters that were not consistent. Only six regions formed the same two clusters; PB, IC, PAG, and PAG-c formed one cluster, while MRN and MRN-c formed another. For output circuits (Figure 4D and Supplementary Figure 5B), six nuclei also gathered into two identical clusters: the SCm, SCm-c, PAG, and PAG-c formed one cluster, while the PB and RR formed another. In addition, a pair of brain regions might display opposing correlations for the input or output. For example, the IC and RR had negative correlations for input to the ChAT^PPN^ neurons but positive correlations for input to the ChAT^LDT^ neurons, which meant that these cholinergic neurons might receive diverse afferent information from the IC and RR. For input and output of the ChAT^PPN^ neurons (Figures 4C,D and Supplementary Figure 5C), nuclei mainly formed different clusters, and some regions even presented opposing correlations, such as the PAG and IC. For ChAT^LDT^ neurons (Figures 4C,D and Supplementary Figure 5D), the SCm, PB, PAG, PAG-c, and RR formed the same cluster in both the input and output circuits.

These results suggest that the ChAT^PPN^ neurons and ChAT^LDT^ neurons have different connection patterns with the same nuclei in input and output circuits.

### ChAT^PPN^ and ChAT^LDT^ Deliver Different Information *via* a Ventral Posterior-Projection Circuit

**FIGURE 5:**
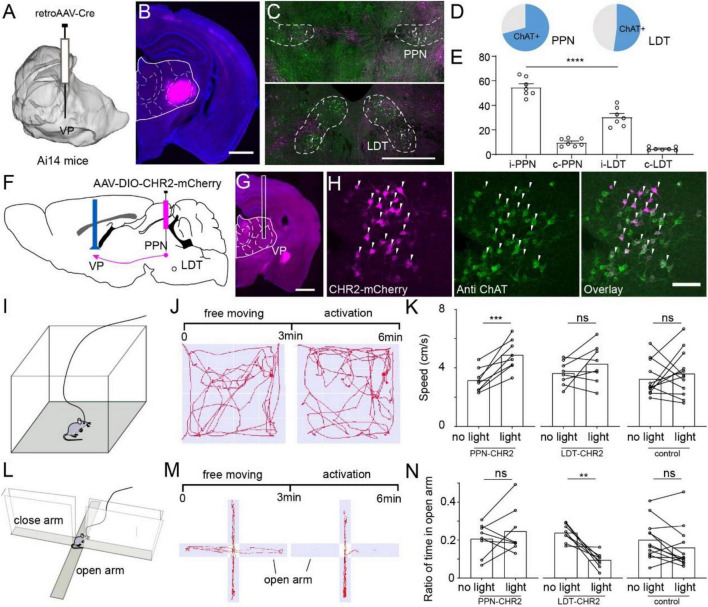
Different roles of ChAT^PPN^ and ChAT^LDT^ projecting to VP. **(A)** Retro-AAV carrying Cre was performed in the VP of Ai14 reporter mice. **(B)** A typical coronal plane of an inject site. **(C)** Immunohistochemical staining of PPN or LDT neurons project to the VP. **(D)** Proportion of ChAT+ neurons projecting from PMT to VP. **(E)** Quantitative statistical proportion of ChAT+ neurons, originating from bilateral PPN and LDT. **(F)** Cre-dependent AAV virus expressing CHR2 and mCherry was injected in the PPN or LDT. **(G)** A typical coronal plane implanted with optical fiber. **(H)** Immunofluorescent staining of CHR2-labeled neurons. **(I)** A schematic diagram of mice in an open field. **(J)** The process of the experiment and typical motion trails of the mice moved in the open field. **(K)** Speed of the mice in the open field. **(L)** A schematic diagram of the mice in an elevated-plus maze. **(M)** The process of the experiment and typical motion trails of the mice moved in the elevated-plus maze. **(N)** The ratio of time that the mice spent in an open arm in the EPM. A scale bar, **(B,G)** 1,000 μm; **(C)** 500 μm; **(H)** 100 μm. Data are shown as mean ± SEM. **(E)** Two-sided *t*-tests, **(K,N)** paired *t*-tests, **p < 0.01, ***p < 0.001, ****p < 0.0001.

The comparison of the input and output atlases revealed unidirectional circuits projected from the ChAT^PPN^ and ChAT^LDT^ neurons to some thalamic nuclei (Figures 4A,B). The VP is a crucial interchange portal in the thalamus that integrates sensorimotor information and is delivered to cortical areas. Our results suggested that the VP received unidirectional cholinergic modulation from the PPN and LDT with quantitative differences (Figure 3F). To confirm the diverse cholinergic projections, we labeled the input of VP. We injected the retro AAV-Cre virus in the VP of Ai14 reporter mice (Figures 5A,B). Three weeks later, dense neurons were present in the PPN and LDT but not in other cholinergic nuclei (Figure 5C and Supplementary Figure 6A). Then, immunofluorescent staining suggested that more than half of the labeled neurons in the PPN and LDT were ChAT positive (PPN, 70.83 ± 2.91%; LDT, 52.13 ± 4.58%) (Figure 5D and Supplementary Figure 6B). Furthermore, we found ChAT-positive neurons mainly located in the ipsilateral to the injection site (i-PPN, 54.90 ± 2.84%; i-LDT, 30.57 ± 2.87%) and a few in the contralateral. Consistent with fiber projection, the PPN sent stronger cholinergic projections to the VP than the LDT (i-PPN vs. i-LDT, *p* < 0.0001; Figures 3F, 5E).

To explore the role of ChAT^PPN^ and ChAT^LDT^ neurons projecting to the VP, we infected and modulated cholinergic fibers *via* optogenetics. First, the ChAT-Cre mice were injected unilaterally with a channelrhodopsin-2 (ChR2)-expressing adeno-associated virus (AAV) (AAV2/9-DIO-hChR2-mCherry) in the PPN or LDT (PPN = LDT = 8 mice). Meanwhile, Cre-dependent mCherry-expressing AAV was injected into the PPN or LDT as a control group (PPN = LDT = 6 mice). The VP of all experimental mice was implanted with optical fibers for photostimulation (Figures 5F,G and Supplementary Figures 6C,D). After the experiment, all the mice were sacrificed, and we certified the labeled cells with immunofluorescence staining (Figure 5H and Supplementary Figure 6E).

First, we evaluated the effects of ChAT^PPN^-VP or ChAT^LDT^-VP circuits on locomotion *via* the open field test (Figure 5I). In the experiments, we gently placed the mice in the center of the open field to explore freely for 2–3 min. Then, we recorded the movement of the mice for 6 min, including 3 min of free movement and, subsequently, 3 min of optical activation (473-nm, 20-Hz, 10-ms light pulses) on the VP (Figure 5J). As shown in Figure 5K, optical activation of ChAT^PPN^-VP circuits increased the speed of mice significantly (paired *t*-test, *p* = 0.001), while there was no obvious change for the LDT and control groups.

Then, we evaluated whether activating the ChAT^PPN^-VP or ChAT^LDT^-VP circuits influenced the anxiety of the mice *via* the elevated plus maze (Figure 5L). Similarly, we placed the mice in the center of the elevated-plus maze with the head toward the closed arms. When the mice tried to explore the open arms, we recorded the movement of the mice for 6 min, including 3 min of free movement and, subsequently, 3 min of optical activation (473-nm, 20-Hz, 10-ms light pulses) on the VP (Figure 5M). Because activating the ChAT^PPN^-VP circuits increased the locomotion of the mice (Figures 5J,K), we first calculated the distance mice moved in the EPM and found that activating the VP circuits did not significantly influence the locomotion of the mice in the EPM (Supplementary Figure 6F). Then, we calculated and compared the time that the mice spent exploring the open arms. As shown in Figures 5M,N, the ratio of time spent in the open arms did not change significantly in the PPN and control groups, but it decreased significantly in the LDT groups (paired *t*-test, *p* = 0.0014).

These results suggest that ChAT^PPN^ sends stronger projections to the VP and enhances the movement of the mice, while activating the ChAT^LDT^-VP circuits induces more anxiety in the mice.

## Discussion

Decoding the long-range connectome of PMT cholinergic neurons would provide indispensable information needed to understand their various and distinct functions. Combining viral tracing with a whole-brain imaging system, we dissected the input and output atlases of the PMT in the whole brain and found that ChAT^PPN^ and ChAT^LDT^ neurons had similar connecting nuclei but with quantitative differences. The neurochemical properties of input neurons revealed modulatory and non-modulatory afferents from the DR, LC, and VTA. Furthermore, we investigated the different roles of cholinergic neurons in the PPN and LDT *via* unidirectional projection to the VP.

### Connectivity of Pontomesencephalic Tegmentum Cholinergic Neurons in Mice

Consistent with previous studies (Wang et al., 2019; Henrich et al., 2020), PMT cholinergic neurons in the mice received inputs from and sent outputs to multiple regions, ranging from cortical areas to the spinal cord. Based on the 3D continuous datasets, we registered the connected atlas of PPN and LDT in the same brain and compared the spatial distribution in specific nuclei, including the BST, CEA, and ZI. We found that the input neurons of the PPN and LDT from the same nuclei were mainly mixed and gathered in certain parts of the nuclei. These results indicate that PMT cholinergic neurons were mainly innervated by some subareas of these nuclei and that they targeted the PPN and LDT equally in connections. The spatial information gave us a better understanding of the input patterns of PMT neurons, and it was difficult to acquire in 2D slices due to the poor Z resolution and differences in sectioning angles between samples. In addition, the systematically mapped input and output atlas allowed us to compare the afferent and efferent connections integrally. In the whole-brain connections, we found that 15 nuclei had strong interactions with ChAT^PPN^ and ChAT^LDT^ neurons simultaneously, but these neurons clustered differently in input and output circuits (Figures 5C,D and Supplementary Figure 5). In particular, some brain regions might display opposing correlations for their input or output. For instance, the IC and RR had negative correlations for input circuits of PPN^ChAT^ but positive correlations for LDT^ChAT^, and the ZI and IC had negative correlations in their input circuits but positive correlations in output circuits. These results suggested that the ChAT^PPN^ and ChAT^LDT^ neurons had similar connections with some brain regions but with different connectivity patterns. In addition, although most nuclei interconnected with them, some regions received abundant cholinergic modulation uniaxially (Figures 4A,B), such as the ATN, VP, GEN, RT, PG, and CBX. They are important targets of ChAT^PPN^ and ChAT^LDT^ and are involved in various functions. These results provide a deeper understanding of interconnections between PMT cholinergic neurons and their connecting nuclei.

As an evolutionarily conserved nucleus, we found that the PMT in the mice and rats contained cholinergic neurons with different numbers (Li et al., 2018; Luquin et al., 2018) but received inputs with similar patterns. The ChAT^PPN^ and ChAT^LDT^ received inputs from similar areas, but the ChAT^PPN^ received stronger afferents from some nuclei related to locomotion, while the ChAT^LDT^ was preferred by limbic cortical areas and pons (Figure 1G; Huerta-Ocampo et al., 2021). Moreover, we confirmed that the ChAT^PPN^ and ChAT^LDT^ in the mice received similar input from the vermis cerebelli and acquired stable afferent information from the spinal cord. Of course, more experiments are needed to compare the similarities and differences in connections between the mice and the rats.

Apart from the cholinergic neurons, the PMT also contains abundant glutamatergic and GABAergic neurons. In the input circuits (Roseberry et al., 2016; Wang et al., 2019; Dautan et al., 2021), we found that the cholinergic and non-cholinergic neurons in the PMT received input information from the same nuclei but were preferred by diverse nuclei. In the output circuits (Henrich et al., 2020; Dautan et al., 2021), cholinergic and glutamatergic neurons in the PPN had different preferences. The cholinergic neurons sent richer fibers to some ascending circuits, especially in the cortical areas and thalamus, which agreed with the results of retrograde tracing from the thalamus (Paré et al., 1988).

### The Interconnection of Neuromodulator Neurons in the Ascending Reticular Activation System

Neuromodulator neurons modulate multiple functions related to arousal, attention or emotion (Sabatini and Tian, 2020), and the interactions between different neuromodulator neurons are important for delivering information and executing functions (Steckler and Sahgal, 1995; Lester et al., 2010). Multiple neuromodulator neurons in the ascending reticular activation system make great contributions to various functions related to arousal (Jones, 2003). Previous studies have demonstrated that PMT cholinergic neurons modulate the activity of neuromodulator-releasing neurons in the DR (Kinoshita et al., 2018), VTA, and SNc (Zhang et al., 2018). In this study, we certified stable monosynaptic inputs from serotonergic neurons in the DR and noradrenergic neurons in the LC to the PMT (Figures 2G–L), which were ignored or failed to be identified in previous studies (Huerta-Ocampo et al., 2021). Previous studies in rats have confirmed that DA neurons in the SNc directly regulate the activity of cholinergic neurons in the PMT (Ryczko et al., 2016) but have difficulty identifying DA neurons in the input circuits of PMT cholinergic neurons (Huerta-Ocampo et al., 2021). Because the modified RV may transsynaptically infect DA neurons weakly (Wall et al., 2013), we traced afferent neurons from the VTA to the PMT *via* multiple retrograde tracers, including the RV, CTB, and FG (Figures 2G,H and Supplementary Figure 3). We found that abundant neurons in the VTA were labeled, but all of them were TH negative. Considering DA neurons in the midbrain, only a few axons project to descending areas (Beier et al., 2015). We speculate that neurons in the VTA receive strong cholinergic modulation from the PMT (Dautan et al., 2016; Xiao et al., 2016) but send direct feedback information mainly *via* non-DA neurons. These results suggested that PMT cholinergic neurons were interconnected with neuromodulatory neurons in the DR and LC but mainly modulated the activity of DA neurons in the VTA uniaxially, which enhanced our knowledge of the organization of the ascending reticular activation system.

### Differential Connections With the Thalamus

The thalamus is an important relay station for sensory information. Various sensory conduction pathways exchange information in the thalamus and then target specific cortical areas (Oh et al., 2014). Studies in multiple species (Sofroniew et al., 1985; Paré et al., 1988; Mesulam et al., 1989; Motts and Schofield, 2010) have certified that the PMT is the main source of acetylcholine for the thalamus. Our results revealed asymmetrical interconnections between the thalamus and PMT; most thalamic nuclei received abundant fibers from the PMT cholinergic neurons, but only several lateral nuclei sent monosynaptic input to the PMT (Figures 4A,B).

The VP is one of most important unidirectional projection paths of PMT cholinergic neurons (Figure 4B) and makes a great contribution to sensorimotor information transmission. The output atlas suggested that ChAT^PPN^ neurons sent richer fibers to the VP, which we confirmed *via* retrograde tracing and immunofluorescent staining (Figures 5A–D). Subsequently, we verified the different roles of two parallel cholinergic circuits and found that activating ChAT^PPN^-VP circuits increased the speed of the mice, while activation of ChAT^LDT^-VP circuits made the mice more anxious (Figures 5F–N). VP neurons are one of the most important input regions of the sensorimotor cortex (Zhao et al., 2020), and we speculate that ChAT^PPN^ and ChAT^LDT^ deliver different sensorimotor information *via* VP circuits. Meanwhile, PMT cholinergic neurons projecting to the striatum send collaterals to the thalamus (Dautan et al., 2014), and we also found that some cholinergic neurons in the PPN or LDT innervated the VP and VTA simultaneously (Supplementary Figure 7). The collaterals of VP projection neurons may also contribute to the functions of cholinergic VP circuits. Furthermore, we observed that the ChAT^PPN^ and ChAT^LDT^ connected similar regions, but the PPN had stronger connections with some nuclei contributing to locomotion, while the LDT interacted tightly with some limbic structures.

In addition, previous studies have certified that PMT cholinergic neurons make great contributions to multiple functions, including auditory sensation (Luo and Yan, 2013), sensorimotor (Muller et al., 2013), and spatial memory (Mitchell et al., 2002), by targeting different thalamic nuclei. We suppose that cholinergic neurons in the PMT deliver different pieces of information by projecting to various thalamic nuclei and that ChAT^PPN^ and ChAT^LDT^ neurons may handle different pieces of information even by targeting the same nuclei.

In conclusion, we mapped the long-range input and output of PMT cholinergic neurons with high resolution systematically and integrally decoded the connectivity patterns of the ChAT^PPN^ and ChAT^LDT^ under the same criteria. We revealed monosynaptic input from serotoninergic neurons in the DR and noradrenergic neurons in the LC. Finally, we decoded diverse cholinergic projections from the PPN and LDT to the VP and verified their different roles in locomotion and anxiety. This study maps the finest connected atlas of the pontine-tegmental cholinergic system and gives us a better understanding of its connection pattern.

## Data Availability Statement

The raw data supporting the conclusions of this article will be made available by the authors, without undue reservation.

## Ethics Statement

The animal study was reviewed and approved by the Institutional Animal Ethics Committee of Huazhong University of Science and Technology.

## Author Contributions

HG and XL conceived and designed the study. PZ performed the experiments and analyzed the data. TJ acquired the continuous whole brain datasets. HW, QS, and AL processed the whole-brain data. XL, HG, and PZ wrote the manuscript. All authors contributed to the article and approved the submitted version.

## Conflict of Interest

The authors declare that the research was conducted in the absence of any commercial or financial relationships that could be construed as a potential conflict of interest.

## Publisher’s Note

All claims expressed in this article are solely those of the authors and do not necessarily represent those of their affiliated organizations, or those of the publisher, the editors and the reviewers. Any product that may be evaluated in this article, or claim that may be made by its manufacturer, is not guaranteed or endorsed by the publisher.
