# Supplementary material for: The Mesoscopic Connectome of the Cholinergic Pontomesencephalic Tegmentum
Source: Front Neuroanat. 2022 May 17;16:843303. doi: 10.3389/fnana.2022.843303 (PMC9152021; doi:10.3389/fnana.2022.843303)
Supplement: Supplementary file 1 [file Data_Sheet_1.docx]

# The Mesoscopic Connectome of Cholinergic Neurons in the mouse Pontomesencephalic Tegmentum

Peilin Zhao^1^, Huading Wang^1^, Anan Li^1, 2, 3^, Guangcai Liu^1^, Yutong Han^1^, Qingtao Sun^2^, Jing Yuan^1, 2^, Tao Jiang^2^, Xueyan Jia^2^, Xiangning Li^1, 2^ *, Hui Gong^1, 2, 3,^ *


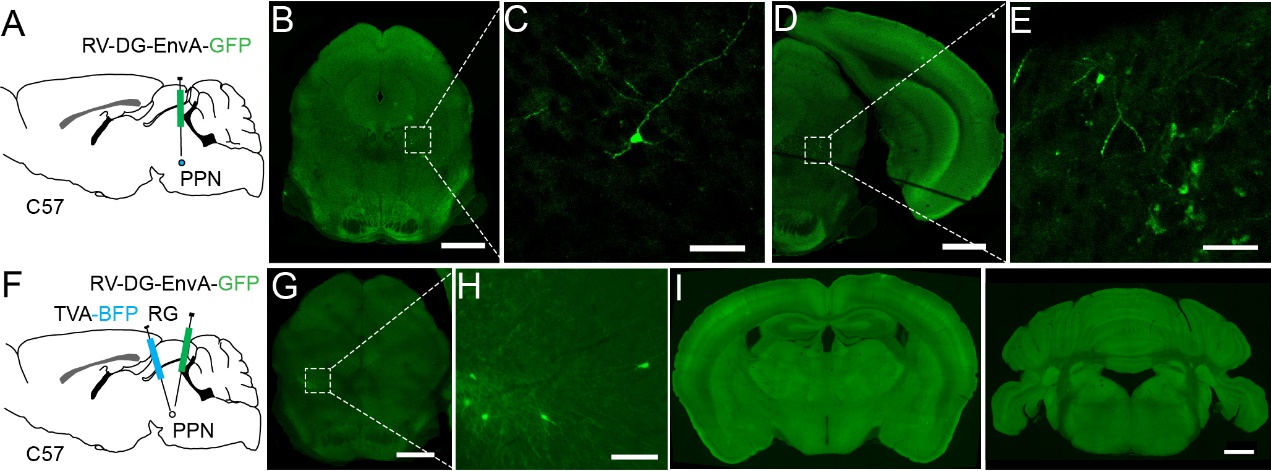


**Supplementary figure S1. The specificity of virus.** **(A)** RV was performed in the PPN of C57 mice directly. **(B) (D)** Several neurons were labeled in the inject site. **(C) (E)** Clear view of the labeled neurons. **(F)** AAV helpers were injected in the PPN of C57 mice and subsequent with modified RV 3 weeks later. **(G)** A few neurons presented in the inject site. **(H)** Clear view of the labeled neurons. (I) No neuron was infected in other input regions. Scale bar, **(B, D, G, I)** 1000 μm; **(C, E, H)** 100μm.


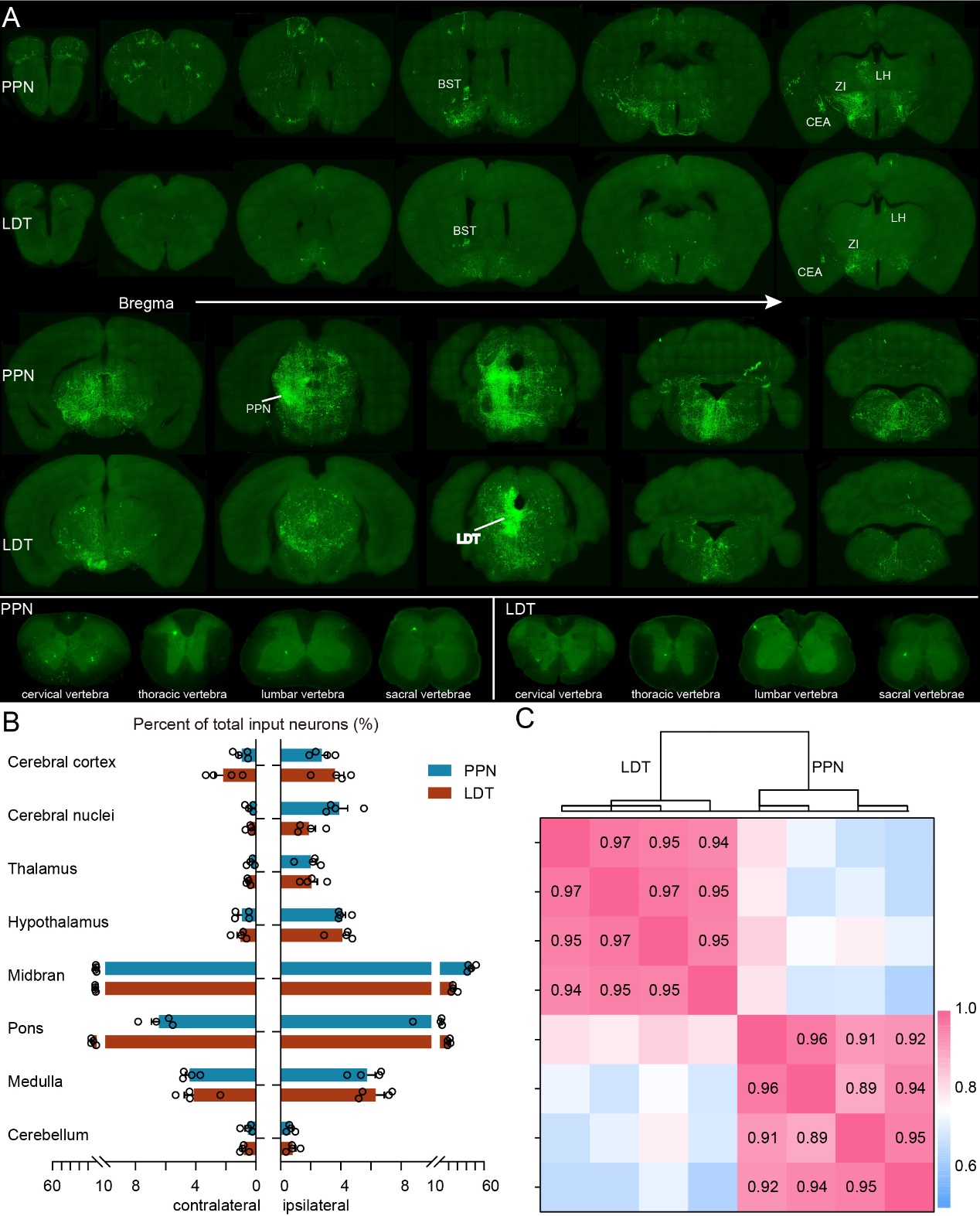


**Supplementary figure S2. Overview of whole-brain input distribution**. **(A)** whole-brain input to PPN and LDT. **(B)** Quantitative statistical proportion of eight input regions to whole-brain input neurons. Left side of vertical axis represents proportion of input neurons in the contralateral hemispheres, and the right side represents ipsilateral hemispheres. Data shown as Mean ± SEM. **(C)** Clustering analysis of all counted input samples according to the Pearson's correlation.


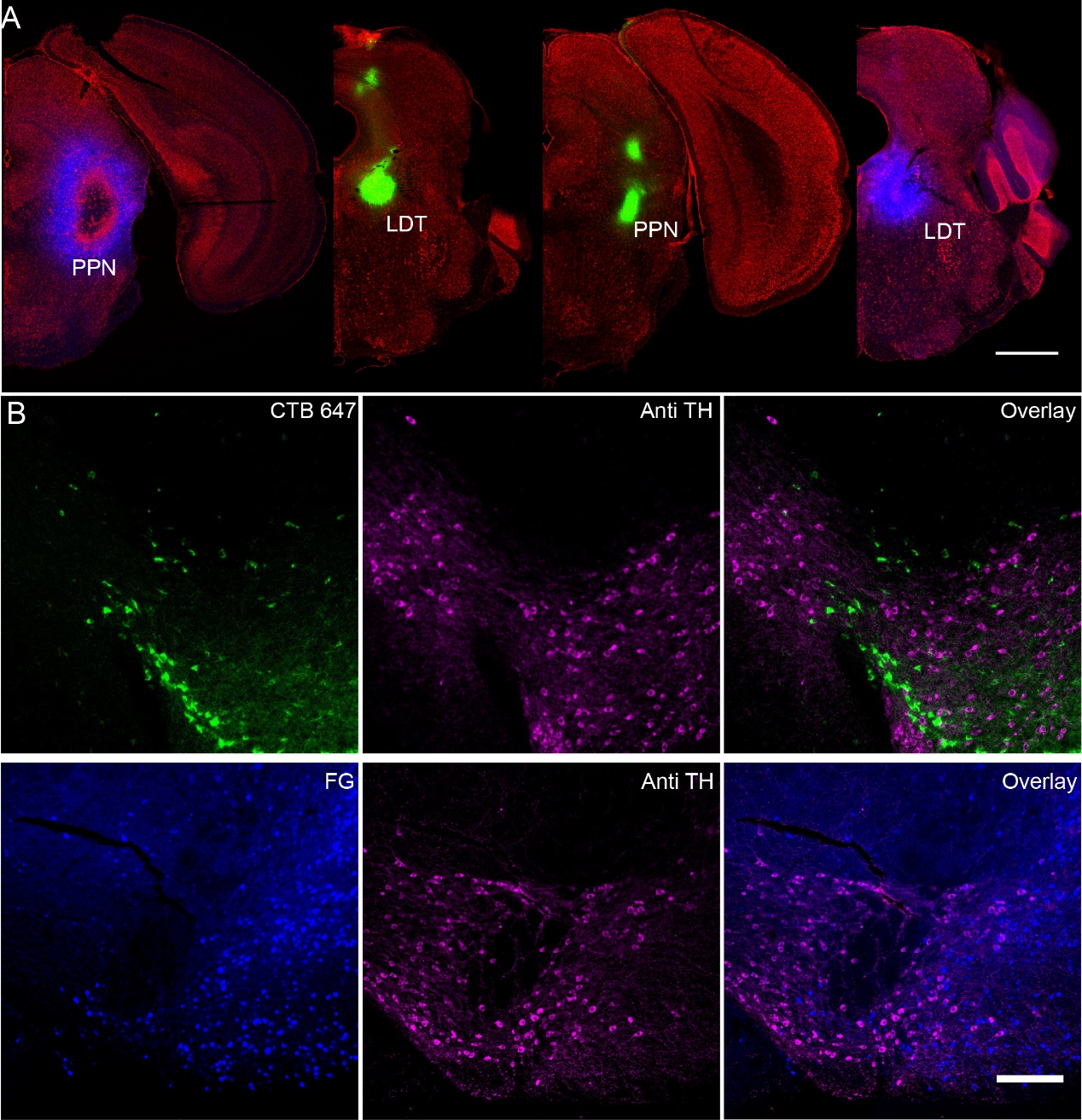


**Supplementary figure S3. Retrograde tracing with CTB and FG.** (A) 75nl 4% FG (blue) or CTB 647 (green) were injected in the PPN or LDT. **(B)** Immunofluorescence staining of labeled in the VTA. All neurons labeled with CTB or FG present TH-negative. Scale bar, **(A)** 1000 μm; **(B)** 200 μm.


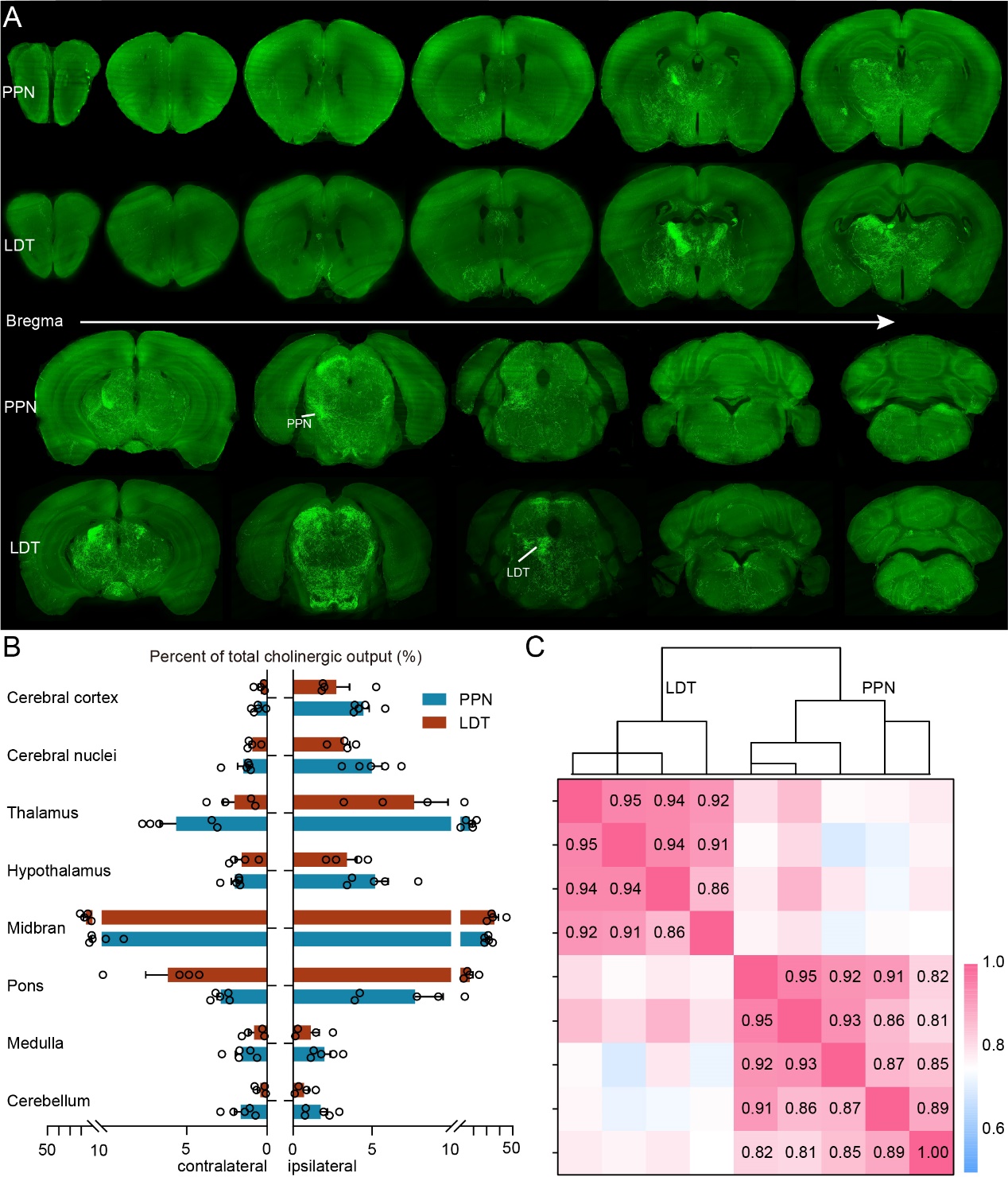


**Supplementary figure S4. Overview of cholinergic fibers in the whole brain.** **(A)** The whole brain projection of cholinergic neurons from PPN and LDT. **(B)** Quantitative statistical proportion of eight main target regions to whole-brain input neurons. Left side of vertical axis represents proportion of input neurons in the contralateral hemispheres, and the right side represents ipsilateral hemispheres. Data shown as Mean ± SEM. **(C)** Clustering analysis of all counted output samples according to the Pearson's correlation.


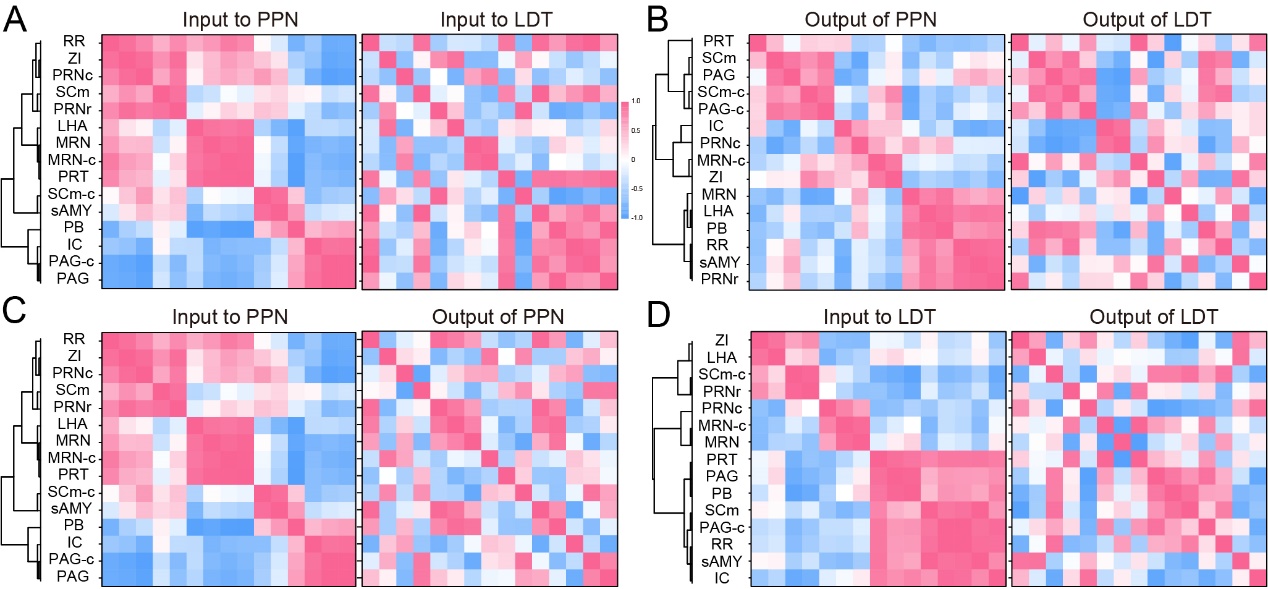


**Supplementary figure S5. The clustering relation of nuclei strongly interconnect with PMT.** **(A)** Clustering relation of input circuits. **(B)** Clustering relation of output circuits. **(C)** Clustering relation of input and output circuits for PPN. **(D)** Clustering relation of input and output circuits for LDT.


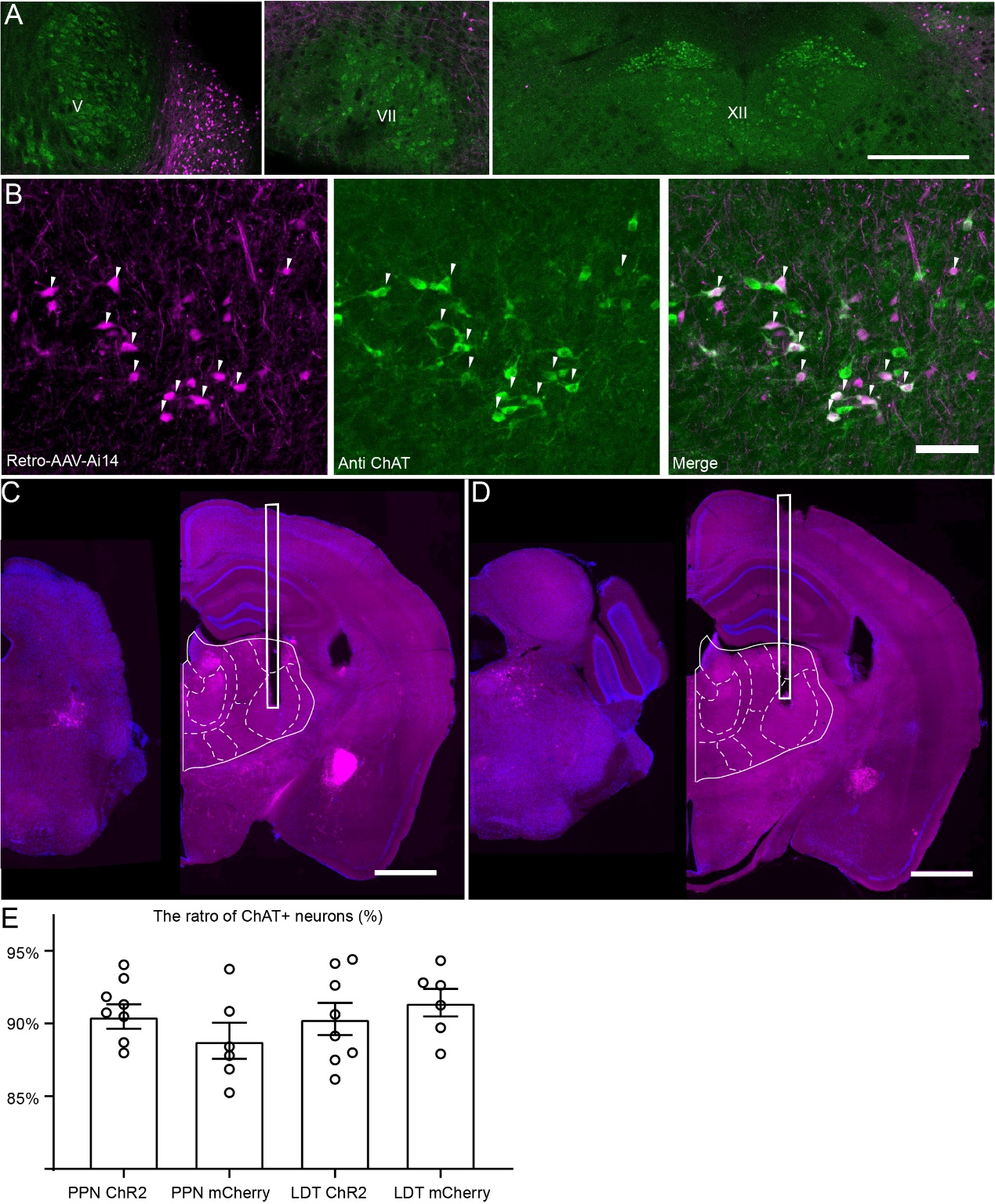


**Supplementary figure S6. Tracing the cholinergic input of VP. (A)** None of cholinergic neurons in the V, VII and XII projected to the VP directly. **(B)** A three-panel presentation with AAV-Ai14, anti-CHAT, and merged, arrows point out ChAT-positive neurons. **(C, D)** Inject site of PPN and LDT and tipical coronal plane of VP embedded with ceramic ferrule. **(E)** Statistical proportion of ChAT+ neurons labeled with ChR2 or mCherry. Scale bar, **(A)** 500 μm; **(B)** 100 μm; **(C, D)** 1000 μm.

**Supplementary Table S1 The abbreviation of brain regions**

| **Abbrevition** | **Full name** |
| --- | --- |
| AI | Agranular insular area |
| AON | Anterior olfactory nucleus |
| ATN | Anterior group of the dorsal thalamus |
| AuD | Auditory areas |
| B | Barrington's nucleus |
| BF | basal forebrain |
| CBN | Cerebellar nuclei |
| CBX | Cerebellar cortex |
| CP | Caudoputamen |
| CS | Superior central nucleus raphe |
| CTXpl | cortical plate |
| CTXsp | cortical subplate |
| DORpm | Thalamus, polymodal association cortex related |
| DORsm | Thalamus, sensory-motor cortex related |
| DR | Dorsal nucleus raphe |
| DTN | Dorsal tegmental nucleus |
| ECT | Ectorhinal area |
| EPI | Epithalamus |
| GENd | Geniculate group, dorsal thalamus |
| GENv | Geniculate group, ventral thalamus |
| GRN | Gigantocellular reticular nucleus |
| GU | Gustatory areas |
| IC | Inferior colliculus |
| ILM | Intralaminar nuclei of the dorsal thalamus |
| IRN | Linear nucleus of the medulla |
| LAT | Lateral group of the dorsal thalamus |
| LDT | Laterodorsal tegmental nucleus |
| LHA | Lateral hypothalamic area |
| LSX | Lateral septal complex |
| LZ | Hypothalamic lateral zone |
| MARN | Magnocellular reticular nucleus |
| MBmot | Midbrain, motor related |
| MBsen | Midbrain, sensory related |
| MBsta | Midbrain, behavioral state related |
| MED | Medial group of the dorsal thalamus |
| MEZ | Hypothalamic medial zone |
| MOp | Primary motor area |
| MOs | Secondary motor area |
| mPFC | medial prefrontal cortex |
| MRN | Midbrain reticular nucleus |
| MY- mot | Medulla, motor related |
| MY-sat | Medulla, behavioral state related |
| MY-sen | Medulla, sensory related |
| NI | Nucleus incertus |
| OLF | olfactory areas |
| ORB | orbital area |
| PAG | Periaqueductal gray |
| PAL | pallidum |
| PALd | Pallidum, dorsal region |
| PALm | Pallidum, medial region |
| PALv | Pallidum, ventral region |
| PARN | Parvicellular reticular nucleus |
| PB | Parabrachial nucleus |
| PCG | Pontine central gray |
| PERI | Perirhinal area |
| PFL | Paraflocculus |
| PG | Pontine gray |
| P-mot | Pons, motor related |
| PPN | Pedunculopontine nucleus |
| PRNc | Pontine reticular nucleus, caudal part |
| PRNr | Pontine reticular nucleus |
| PRT | Pretectal region |
| P-sat | Pons, behavioral state related |
| P-sen | Pons, sensory related |
| PVR | periventricular region |
| PVZ | periventricular zone |
| RR | Midbrain reticular nucleus, retrorubral area |
| RT | Reticular nucleus of the thalamus |
| SAG | Nucleus sagulum |
| sAMY | Striatum-like amygdalar nuclei |
| SCm | Superior colliculus, motor related |
| SCs | Superior colliculus, sensory related |
| SLC | Subceruleus nucleus |
| SLD | Sublaterodorsal nucleus |
| SNr | Substantia nigra, reticular part |
| STR | striatum |
| STRv | Striatum ventral region |
| Tea | Temporal association areas |
| TRN | Tegmental reticular nucleus |
| VENT | Ventral group of the dorsal thalamus |
| VISC | Visceral area |
| VNC | Vestibular nuclei |
| VP | Ventral posterior complex of the thalamus |
| VTA | Ventral tegmental area |
| ZI | Zona incerta |

**Supplementary Table S2 Detail P values of significance analysis**

| PPN VS LDT | Input (P values) | | PPN VS LDT | Output (P values) | |
| --- | --- | --- | --- | --- | --- |
|  | Ipsilateral | Contralateral |  | Ipsilateral | Contralateral |
| ORB | ns | ns | OLF | ns | ns |
| mPFC | 0.0436 | ns | STRv | 0.0474 | ns |
| Mos | 0.0292 | ns | CP | 0.0369 | ns |
| CP | 0.0082 | ns | sAMY | ns | ns |
| sAMY | ns | ns | LSX | ns | ns |
| VENT | ns | ns | PALv | ns | ns |
| EPI | ns | ns | PALd | 0.0469 | ns |
| MEZ | 0.0294 | ns | PALm | ns | ns |
| ZI | ns | ns | RT | 0.0297 | ns |
| LHA | ns | ns | VP | 0.0110 | 0.0140 |
| DR | ns | ns | VENT | 0.0082 | 0.0107 |
| PPN | <0.0001 | ns | GENd | ns | ns |
| IC | ns | ns | MED | 0.0470 | ns |
| RR | ns | ns | LAT | 0.0335 | 0.0038 |
| CUN | 0.0043 | 0.0370 | ILM | 0.0225 | ns |
| VTA | 0.4290 | ns | GENv | ns | ns |
| SNr | 0.0418 | ns | ATN | ns | ns |
| SCm | 0.0017 | ns | MEZ | ns | ns |
| PRT | 0.4387 | ns | ZI | 0.0055 | 0.0423 |
| PAG | ns | ns | LHA | ns | ns |
| MRN | 0.0104 | ns | IPN | ns | ns |
| PB | ns | ns | PPN | 0.0001 | ns |
| SLD | 0.0407 | ns | SAG | 0.0206 | ns |
| PRNr | ns | ns | SCs | 0.0094 | 0.0137 |
| NI | 0.0115 | 0.0166 | IC | ns | ns |
| LDT | 0.0039 | 0.0102 | RR | ns | ns |
| CS | 0.0124 | 0.0155 | CUN | ns | ns |
| DTN | 0.0160 | 0.0022 | VTA | ns | ns |
| B | 0.0048 | 0.0079 | SCm | 0.0002 | 0.0016 |
| PRNc | 0.0371 | 0.0032 | PRT | ns | ns |
| PCG | <0.0001 | 0.0001 | PAG | ns | ns |
| PARN | 0.0102 | 0.0283 | MRN | 0.0332 | ns |
| MARN | ns | ns | PB | ns | ns |
| IRN | ns | ns | SLD | 0.0246 | ns |
| GRN | ns | ns | SLC | ns | ns |
| VNC | ns | ns | PRNr | ns | ns |
| PGRN | ns | ns | CS | 0.0211 | 0.0192 |
| CBN | ns | ns | PG | ns | ns |
|  |  |  | DTN | 0.0114 | ns |
|  |  |  | B | 0.0364 | ns |
|  |  |  | TRN | ns | ns |
|  |  |  | PRNc | ns | ns |
|  |  |  | PCG | 0.0001 | 0.0002 |
|  |  |  | CBX | 0.0408 | 0.0083 |
